# Impact of COVID-19 on inpatient care related to skin conditions

**DOI:** 10.3389/fmed.2023.1123989

**Published:** 2023-03-02

**Authors:** Karolina Kalanj, Ante Orbanić, Mirta Peček, Stjepan Orešković, Rick Marshall, Mirna Šitum

**Affiliations:** ^1^Department of Medical Oncology, Clinic of Oncology, Clinical Hospital Center, Zagreb, Croatia; ^2^Andrija Štampar School of Public Health, School of Medicine, University of Zagreb, Zagreb, Croatia; ^3^City Pharmacy Zagreb, Zagreb, Croatia; ^4^School of Medicine, University of Zagreb, Zagreb, Croatia; ^5^Epidemiologist and Independent Consultant in Health System Funding Models, Eaglehawk Neck, TAS, Australia; ^6^University Hospital Centre Sestre Milosrdnice, Zagreb, Croatia; ^7^School of Dental Medicine, University of Zagreb, Zagreb, Croatia

**Keywords:** COVID-19, AR-DRG, data transparency, dermatology, inpatient care, pandemic, patient classification system, health system response

## Abstract

**Background:**

The COVID-19 pandemic significantly affected our society and healthcare system. This study aims to evaluate the effects of COVID-19 on the number of hospitalized patients with dermatological diseases in Croatia, as well as the number of these patients treated surgically and conservatively, before (2017–2019) and during the pandemic (2020–2021).

**Materials and methods:**

This is a retrospective, comparative study of the hospital admission rate for patients with skin, subcutaneous tissue, and breast disorders both before and during the pandemic. This study used data from the Croatian Institute of Public Health (CIPH) and the Croatian Health Insurance Fund (CHIF). Inpatient data for the CHIF data collection were categorized using the Australian Refined Diagnosis Related Groups (AR-DRGs). All hospital admissions for dermatology patients at all non-specialized hospitals in Croatia were tracked during two periods, before (2017–2019) and during the pandemic (2020–2021).

**Results:**

The average number of dermatology patients in all hospitals fell by 29% during the pandemic. The overall number of dermatological patients admitted to hospitals fell by 32% in 2020 and by 26% in 2021 when compared to the number of patients admitted each year on average over the preceding 3 years. Additionally, there was an average 22% fall in surgical procedures performed during the pandemic. The only exception for surgical procedures is Major Breast Reconstruction for which is noted an increase, as also for Malignant Breast Disorders, Minor Complexity and Major Skin Disorders, Minor Complexity in a group of non-surgically treated patients.

**Conclusion:**

Examining the all consequences of the Croatian dermatological patient care interruption will require more investigation. Reduced access to medical care during the pandemic is anticipated to lead to later illness diagnosis, a later start to treatment, a poorer disease prognosis, as well as higher medical expenditures.

## Introduction

In Wuhan, China, as of 31 December, 2019, several cases of pneumonia with an unclear origin had been documented. The severe acute respiratory syndrome coronavirus 2 (SARS-CoV2) pathogen was discovered, and the SARS-CoV2 infection outbreak was given the term coronavirus disease 2019 (COVID-19) by the World Health Organization (WHO). A rapidly spreading new coronavirus (COVID-19) outbreak was classified as a worldwide pandemic by WHO on 11 March, 2020 (1).

European countries have put measures in place to contain their epidemics in reaction to the rising numbers of illnesses and fatalities and to safeguard health systems. These extensive population health interventions vary by the nation but they have included social isolation, border closures, school closings, measures to isolate symptomatic people and their contacts, and population lockdowns except for necessary internal transit (2). Croatia’s response to COVID-19 largely replicated the policies of other European nations.

During the pandemic, significant changes in the clinical practice of several medical specialties have been seen. The quality and length of visits to dermatology departments may be impacted by the use of personal protective equipment (PPE) and social isolation: patients tend to show the affected part of the body, facial protections can interfere with dermoscopy, and elderly patients with hearing impairments struggle to understand the prescription because they cannot see the label. Both medical professionals and patients are hesitant to examine the mouth cavity and/or assess face abnormalities. Physicians cannot advise simply stopping the use of PPE, although it has been shown that facial protections cause occlusion and, as a result, a moist and warm microenvironment, which can either cause or worsen facial dermatoses. As a result of facial skin damage causing pruritus, which can lead to the user scratching their face or removing their mask, and decreasing the efficacy of PPE, treating these dermatoses may help reduce COVID-19 infection. However, if the work activity permits it, a surgical mask can be advised in place of an N95 mask (3, 4). Elective treatments were generally delayed as a result of hospital care reorientation and claim re-prioritization, which prioritized the treatment of patients admitted for COVID-19 and non-COVID-19 emergencies. Because of this, some patients went to private healthcare providers. Private practitioners also faced significant financial hardships during the pandemic outbreak, particularly during the lockdown (5).

The use of virtual consultation techniques significantly increased during the pandemic outbreak. Additionally, the popularity of virtual consultations has dramatically expanded and is now a successful model for offering patients needed contactless therapy (6). In comparison to phone-based consultations, patients who used video-based consultations reported higher levels of satisfaction, but not considerably. While older patients stated that they would prefer a face-to-face visit, the majority of patients reporting satisfaction with the teledermatology services, including video- and phone-based consultations, were younger patients with acne or chronic skin conditions (psoriasis and hidradenitis suppurativa). Despite the high level of satisfaction, the majority of patients did not find a teledermatology consultation to be as satisfying as a face-to-face visit. There are still significant challenges, including privacy issues, medicolegal concerns, a lack of a formal, accepted method of informed consent during such consultations, and a lack of specific, secure online teledermatology platforms. To enhance these services and boost both the safety and satisfaction of teledermatology consultations for patients and dermatologists, authoritative guidelines and recommendations are urgently required (7).

Many people curtailed or ceased their usual activities, such as going to hospitals or outpatient clinics, to avoid gatherings. The likely cause of the decline in outpatient visits is the avoidance of hospitalization owing to the COVID-19 threat. Older age, orientation, anxiety, avoiding crowded areas, increasing hand washing frequency, and wearing protective masks were all major factors that contributed to the non-attendance at scheduled appointments in outpatient clinics in pandemic (8).

Dermatology practice during the pandemic has been similarly affected by the aforementioned factors (9). Due to flexible work hours and dermatologists’ involvement in COVID-19-related duties during this time, the number of patients visiting dermatology outpatient clinics was dramatically reduced (10). The main reported changes in dermatologists’ standard clinical practice during the COVID-19 outbreak included a decrease in face-to-face consultations, extensive use of teledermatology, uncertainty regarding the COVID-related risks of immunosuppressive/immunomodulating systemic therapies, and a decline in the frequency of cosmetic procedures (9). Dermatologists also played a key role in the pandemic and post-pandemic period with the help of teledermatology in recognizing post-COVID-19 and post-COVID-19 vaccination reactions. Unspecific injection-site reactions, type I hypersensitivity reactions (urticaria, angioedema, and anaphylaxis), type IV hypersensitivity reactions, including delayed large local skin lesions (“COVID arm”); inflammatory reactions in dermal filler or previous radiation sites or even old BCG scars, morbilliform and erythema multiforme-like rashes, as well as autoimmune-mediated skin findings (leukocytoclastic vasculitis, lupus erythematosus, and immune thrombocytopenia), pityriasis rosea-like rashes and reactivation of herpes zoster have been reported following COVID-19 vaccination (11).

This study aims to assess the direct effects of COVID-19 on the number of hospitalized patients with dermatological diseases, as well as the number of patients treated surgically and conservatively, at the level of secondary and tertiary health care, in the period before (2017–2019) and during the pandemic (2020–2021).

## Materials and methods

### Study design and data sources

Data was collated from the Croatian Health Insurance Fund (CHIF) and the Croatian Institute of Public Health (CIPH) databases, which are both publicly accessible (12). The Australian AR-DRG system, upon which the Croatian DRG system is based, uses a modified version of the ICD-10AM and ICD-10 categories for diagnosis coding and Australian Classifications of Health Interventions (ACHI) for procedure coding. The AR-DRG version 5.2, which categorizes episodes of care into 671 DRG classes, is the basis for the DRG grouping method (13). According to AR-DRG structure, the 23 Major Diagnostic Categories (MDC)^1^ broadly represent the patient classification of inpatient episodes of care mostly based on the main diagnose, which is the primary reason for admission. A specific bodily system or etiology is represented by each MDC, and the system is aligned with the ICD10 classification structure. In this research MDC-09 group was observed as this category represents Skin, Subcutaneous Tissue, and Breast diseases (14).

The observed period was from the year 2017 to the year 2021 for all hospital admissions for dermatological patients in the MDC-09 group in all non-specialized acute hospitals in Croatia. These hospitals care for 3.9 million patients, which is 96% of all inpatient activity. In total 11 hospitals on the tertiary level and 28 hospitals on the secondary level were observed. Hospital admissions were calculated as total admission and separated into two groups; surgical and non-surgical admissions. Each group was divided into respective AR-DRG groups based on the indication of patient admission and operating room procedures, and accordingly, changes in patient admission before and during the COVID-19 pandemic were monitored.

Given that the data utilized were fully anonymized and made available as public information from CHIF and the CIPH regulated by Croatian data protection laws, our study did not require informed consent or ethical approval.

### Data and statistical analysis

The average number of inpatient cases was calculated across 3 years (2017–2019) and 2 years (2020–2021). Thereafter, the incidence rate was computed as the average number of cases divided by the average total population over the specific period (2017–2019 and 2020–2021). Finally, the% change in incidence rate was derived as a change in incidence rate between each of the two periods divided by the incidence rate in the earlier period. The 2-by-2 Chi-square test was conducted to compare the incidence rate between the two periods.

The incidence rate ratio (IRR) was estimated as a ratio of two rates. The 95% confidence intervals were calculated based on the Wald method which examines whether the IRR was equal to one. All statistical analyses were performed using R (R Core Team, Austria) (15).

In order to understand the impact of different measures had been put in place on inpatient activity in 2020, for each MDC group hospital admissions data were summarized into three time periods: April lockdown phase number of admissions (COVID-19 1st wave), and average monthly admission rate averaged over the May to August (period of easing restrictions) and September to December periods, respectively (COVID-19 2nd wave).

The distribution of admissions by MDC was compared over the three periods (April Lockdown Phase, May to August 2020, and September to December 2020) using a contingency table and a chi-square test of association between MDC and period.

For each MDC group and each period, a risk of an MDC admission during a specific period was calculated as the proportion of admissions in that MDC group from among all admissions during that period. A risk ratio (or relative risk) for comparing MDC admission risk over two consecutive periods is defined as MDC risk in the second period divided by MDC risk in the first period. Risk ratios and the associated 95% confidence intervals were estimated for each MDC and for both the comparison between admissions in the April Lockdown Phase vs. those in the May to August 2020 period and for the May to August 2020 vs. the September to December 2020 period. In addition to the unadjusted 95% confidence intervals for risk ratios, Holm-multiplicity adjusted *p*-values (16) and the unadjusted/raw *p*-values (for testing the hypotheses of MDC risk ratios being equal to one) were also provided.

Statistical analyses were performed using SAS software (version 9.4). *P*-values of 0.05 or less (two-tailed) were considered to be statistically significant.

## Results

The average number of dermatological patients in all hospitals during the pandemic (2020–2021) was 13,800, of which 8,594 were treated at the tertiary health care level, compared to pre-pandemic (2017–2019) when the average number of patients in all hospitals was 19,959, of which 12,542 were treated at the tertiary health care level. The rate change is −29%, similar for both health care levels (−29%, −28%, *p* < 0.0001, respectively).

For 2020, the total number of hospitalized dermatological patients compared to the average number of patients for the previous 3 years in all hospitals decreased by 32% (an average of 19959 patients between 2017 and 2019 to 13,467 patients in 2020). In 2020, there were 8,338 patients treated at the tertiary and 5,129 patients at the secondary health care level, compared to 2017–2019 when at the tertiary level were 12,542 patients, and at the secondary level 7,417 patients. The rate change was similar at both tertiary and secondary healthcare levels (−33%, −30%, *p* < 0.0001, respectively).

For 2021, the total number of hospitalized dermatological patients compared to the average number of patients for the previous 3 years in all hospitals decreased by 26% (an average of 19,959 patients between 2017 and 2019 to 14,134 patients in 2020). In 2021, there were 8,850 patients treated at the tertiary and 5,284 patients at the secondary health care level, compared to 2017–2019 when at the tertiary level were 12,542 patients, and at the secondary level 7,417 patients. The rate change was similar at both tertiary and secondary healthcare levels (−26%, −25%, *p* < 0.0001, respectively).

Table 1 compares the average number of total admissions during the pre-pandemic (2017–2019) and pandemic years (2020–2021, 2020, and 2021, respectively).

**TABLE 1:** Comparison of total hospital admissions during pre-pandemic (2017–2019) and pandemic (2020–2021, 2020, and 2021, respectively).

Periods		Number of admissions	% Rate change compared to period 2017–2019	*P*
2020–2021	Tertiary hospitals	8,594	−29%	<0.0001
	Secondary hospitals	5,207	−28%	
	Average all hospitals	13,800	−29%	
2020	Tertiary hospitals	8,338	−33%	<0.0001
	Secondary hospitals	5,129	−30%	
	Average all hospitals	13,467	−32%	
2021	Tertiary hospitals	8,850	−26%	<0.0001
	Secondary hospital	5,284	−25%	
	Average all hospitals	14,134	−26%	

CHIF; Authors calculation.

The dermatological patients were treated surgically and conservatively at both healthcare levels. Based on AR-DRG structure, surgical cases are presented with the following AR-DRG groups: J01Z-J14Z^2^ and patients treated conservatively with J60A-J68B groups.^3^

**FIGURE 1:**
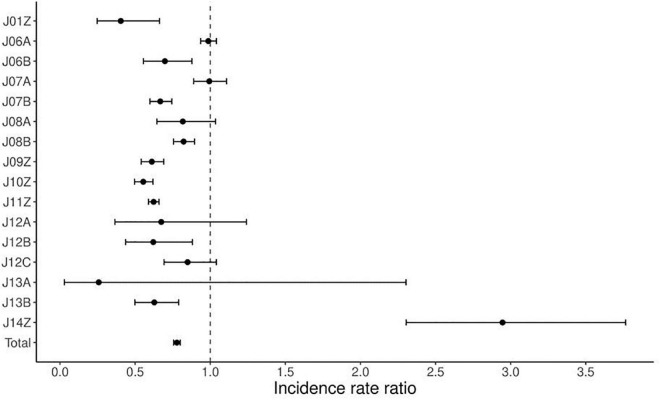
Incidence rate ratio (IRR) for surgical procedures during the pandemic (2020–2021) compared to pre-pandemic (2017–2019).

Table 2 compares the average number of surgical procedures done during the pre-pandemic (2017–2019) and pandemic (2020–2021). An average decrease of 22% was noted. The decrease greater than average is related to J01Z by 60% (*p* = 0.0003), J06Bby 30% (*p* = 0.0025), J07B by 33% (*p* < 0.0001), J09Z by 39% (*p* < 0.0001), J10Z by 45% (*p* < 0.0001), J11Z by 38% (*p* < 0.0001), J12A by 33% (*p* = 0.2612), J12B by 38% (*p* = 0.0092), J13A by 74% (*p* = 0.3889), J13B by 37% (*p* = 0.0001). A decrease less than average was observed in: J06A of 1% (*p* = 0.6427), J07A by 1% (*p* = 0.9262), J08A by 18% (*p* = 0.1065), J08B by 18% (*p* < 0.0001), J12C by 15% (*p* = 0.1271). The decrease at the tertiary level was 25% and at the secondary 17%. There was an increase of 206% for J14Z at the tertiary level, compared to 67% at the secondary. A greater decrease at the tertiary level was for J08B, J09Z, J10Z, and J11Z compared to the secondary level. Figure 1 shows the corresponding IRRs calculated for every procedure.

**TABLE 2:** Comparison of surgical procedures done during pre-pandemic (2017–2019) and pandemic (2020–2021).

AR-DRG	2017–2019 Average tertiary hospitals	2017–2019 Average secondary hospitals	2017–2019 Average all hospitals	2020–2021 Average tertiary hospitals	2020–2021 Average secondary hospitals	2020–2021 Average all hospitals	% Rate change tertiary hospitals	% Rate change secondary hospitals	% Rate change total hospitals	*P*
J01Z	50	5	56	20	2	22	−59%	−59%	−60%	0,0003
J06A	2,087	684	2,771	1,978	678	2,656	−2%	2%	−1%	0,6427
J06B	114	66	180	56	66	122	−49%	3%	−30%	0,0025
J07A	418	230	647	430	194	624	6%	−13%	−1%	0,9262
J07B	454	364	818	272	258	530	−38%	−27%	−33%	<0.0001
J08A	103	52	155	76	47	123	−24%	−7%	−18%	0,1065
J08B	730	482	1,212	548	420	968	−23%	−10%	−18%	<0.0001
J09Z	304	378	681	170	235	404	−42%	−36%	−39%	<0.0001
J10Z	671	229	900	314	170	484	−52%	−24%	−45%	<0.0001
J11Z	2,100	1,072	3,171	1,122	796	1,918	−45%	−24%	−38%	<0.0001
J12A	13	13	26	7	10	17	−45%	−21%	−33%	0,2612
J12B	38	45	83	22	28	50	−40%	−36%	−38%	0,0092
J12C	82	122	205	56	113	169	−30%	−5%	−15%	0,1271
J13A	3	1	4	1	0	1	−66%	−100%	−74%	0,3889
J13B	121	71	192	76	42	117	−35%	−39%	−37%	0,0001
J14Z	78	8	86	232	13	246	206%	67%	195%	<0.0001
	7,366	3,822	11,187	5,380	3,072	8,451	−25%	−17%	−22%	<0.0001

CHIF; Authors calculation.

**FIGURE 2:**
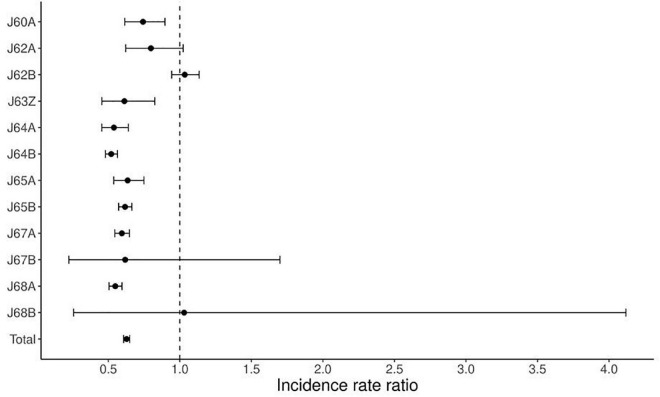
Incidence rate ratio (IRR) for non-surgical treatment during the pandemic (2020–2021) compared to pre-pandemic (2017–2019).

Table 3 shows changes in the average numbers of non-surgically treated patients during pre-pandemic (2017–2019) and pandemic (2020–2021). During the pandemic, there was an average decrease of 37% (from 8,770 to 5,350 patients), by 36% at the tertiary level and 39% at the secondary, respectively. The decrease greater than average is related to the group of diagnoses: J63Z by 39% (*p* = 0.0015), J64A by 46% (*p* < 0.0001), J64B by 48% (*p* < 0.0001), J65A by 36% (*p* < 0,0001), J65B by 38% (*p* < 0.0001), J67A by 41% (*p* < 0.0001), J67B by 38% (*p* = 0.4894), J68A by 45% (*p* < 0.0001). A decrease less than average was noted in groups: J60A by 26% (*p* = 0.0022), J62A by 20% (*p* = 0.0855). The average increase of 3% was related to J62B (*p* = 0.488), and J68B (*p* > 0.9999). An average decrease was greater at the secondary level (39% vs. 36%), except for these group of diagnoses: J64A, J64B, J64B. Figure 2 shows the corresponding IRRs calculated for every group of diagnoses.

**TABLE 3:** Comparison of non-surgical treatment done during pre-pandemic (2017–2019) and pandemic (2020–2021).

AR-DRG	2017–2019 Average tertiary hospitals	2017–2019 Average secondary hospitals	2017–2019 Average all hospitals	2020–2021 Average tertiary hospitals	2020–2021 Average secondary hospitals	2020–2021 Average all hospitals	% Rate change tertiary hospitals	% Rate change secondary hospitals	% Rate change total hospitals	*P*
J60A	110	148	258	68	118	186	−36%	−18%	−26%	0,0022
J62A	101	41	142	80	30	110	−18%	−25%	−20%	0,0855
J62B	633	260	893	734	164	897	19%	−35%	3%	0,488
J63Z	38	78	116	22	46	69	−40%	−39%	−39%	0,0015
J64A	218	164	382	102	98	200	−52%	−38%	−46%	<0.0001
J64B	790	953	1,743	374	507	881	−51%	−45%	−48%	<0.0001
J65A	109	260	368	69	158	227	−35%	−37%	−36%	<0.0001
J65B	796	1,024	1,820	446	644	1,090	−42%	−35%	−38%	<0.0001
J67A	979	434	1,413	566	250	816	−40%	−41%	−41%	<0.0001
J67B	6	4	10	4	2	6	−31%	−49%	−38%	0,4894
J68A	1,392	229	1,621	746	118	864	−45%	−47%	−45%	<0.0001
J68B	2	2	4	3	0	4	54%	−100%	3%	>0.9999
	5,174	3,597	8,770	3,214	2,135	5,350	−36%	−39%	−37%	<0.0001

CHIF; Authors calculation.

**FIGURE 3:**
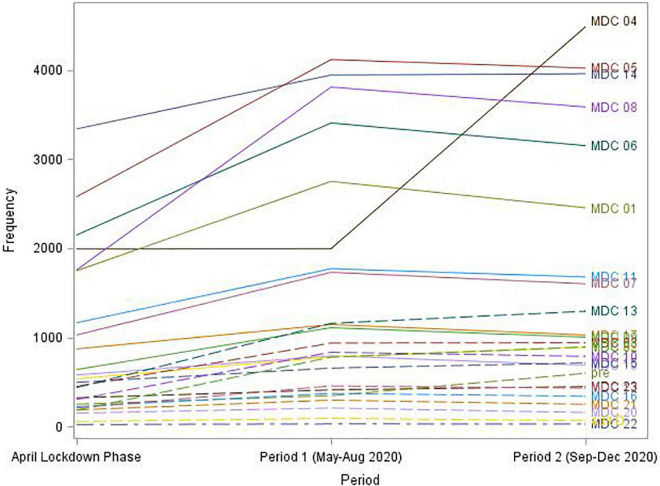
Average monthly admission rate (“Frequency”) by period and major diagnostic category.

Figure 3 portrays a trend in the average monthly admission rate over the three time periods in 2020 grouped by MD categories. For all but one MDC, there was a considerable increase in activity after the April lockdown phase followed by a slight decrease in the average monthly admission rate from period 1 (May to August) to 2 (September to December). A noticeably different trend is demonstrated for the MDC 04 (Respiratory System) group, where the average monthly admission rate was relatively stable over the April and May to August phases but then evidenced a substantial increase in activity during the September to December period.

A more insightful view of the admissions data is provided in Table 4, where the number of monthly admissions for each MDC group and period are shown as both absolute and relative frequencies. In other words, each cell of the table also includes, in addition to the number of admissions (“Frequency”), the percent that each MDC represents of the total admissions during a specific period (“Row percentage”) and the percent that each period represents of the total admissions associated with a specific MDC (“Column percentage”). For example, in the MDC 04 group (Respiratory system), the average monthly admission rate for the May-Aug period is almost the same as in the April lockdown phase (1994 and 1991, respectively). However, a more insightful and reasonable interpretation is available from the comparison of MDC 04 row percentages over the two periods. The MDC 04 relative admission rate, expressed as the percent of total period admissions was relatively high (9.2%) during the April lockdown phase, decreased to 5.88% during the Summer period, and increased again to 12.65% in the Fall. In other words, patients admitted to a hospital during the Sep-Dec period were more than two times as likely to be categorized in the MDC 04 (respiratory system) group as those admitted during the May to August period of 2020.

**TABLE 4:** Contingency table of associations between period and major diagnostic categories.

Period	MDC												
Frequency Row Pct Col Pct	Pre	MDC 01	MDC 02	MDC 03	MDC 04	MDC 05	MDC 06	MDC 07	MDC 08	MDC 09	MDC 10	MDC 11	MDC 12
April Lockdown Phase	250 1.15 20.96	1,746 8.07 25.13	183 0.85 9.87	449 2.07 19.32	1,991 **9.20** 23.50	2,577 11.90 24.05	2,148 9.92 24.68	1,026 4.74 23.55	1,756 8.11 19.19	638 **2.95** 23.22	303 1.40 15.76	1,163 5.37 25.22	208 0.96 19.03
Period 1 (May to August 2020)	346 1.02 29.00	2,750 8.11 39.57	778 2.29 41.96	936 2.76 40.28	1,994 **5.88** 23.54	4,117 12.14 38.42	3,406 10.05 39.13	1,729 5.10 39.69	3,809 11.23 41.63	1,108 **3.27** 40.32	833 2.46 43.32	1,771 5.22 38.40	455 1.34 41.63
Period 2 (September to December 2020)	597 1.68 50.04	2,453 6.91 35.30	893 2.52 48.17	939 2.65 40.40	4,487 **12.65** 52.96	4,022 11.34 37.53	3,151 8.88 36.20	1,601 4.51 36.75	3,585 10.11 39.18	1,002 **2.82** 36.46	787 2.22 40.93	1,678 4.73 36.38	430 1.21 39.34
Total	1,193 1.31	6,949 7.63	1,854 2.04	2,324 2.55	8,472 9.31	10,716 11.77	8,705 9.56	4,356 4.79	9,150 10.05	2,748 3.02	1,923 2.11	4,612 5.07	1,093 1.20
**Period**	**MDC**												
**Frequency** **Row Pct** **Col Pct**	**MDC 13**	**MDC 14**	**MDC 15**	**MDC 16**	**MDC 17**	**MDC 18**	**MDC 19**	**MDC 20**	**MDC 21**	**MDC 22**	**MDC 23**	**Error** **DRGs**	**Total**
April Lockdown Phase	437 2.02 15.14	3,339 15.42 29.70	495 2.29 26.57	223 1.03 23.90	870 4.02 28.62	534 2.47 24.24	578 2.67 28.10	149 0.69 29.04	186 0.86 25.48	22 0.10 28.57	322 1.49 27.27	56 0.26 26.42	21,649 23.78
Period 1 (May to August 2020)	1,157 3.41 40.08	3,944 11.63 35.09	655 1.93 35.16	371 1.09 39.76	1,144 3.37 37.63	780 2.30 35.41	792 2.34 38.50	207 0.61 40.35	295 0.87 40.41	28 0.08 36.36	411 1.21 34.80	91 0.27 42.92	33,907 37.25
Period 2 (September to December 2020)	1,293 3.64 44.79	3,958 11.16 35.21	713 2.01 38.27	339 0.96 36.33	1,026 2.89 33.75	889 2.51 40.35	687 1.94 33.40	157 0.44 30.60	249 0.70 34.11	27 0.08 35.06	448 1.26 37.93	65 0.18 30.66	35,476 38.97
Total	2,887 3.17	11,241 12.35	1,863 2.05	933 1.02	3,040 3.34	2,203 2.42	2,057 2.26	513 0.56	730 0.80	77 0.08	11,81 1.30	212 0.23	91,032 100.00

On the other hand, in the MDC 05 group, the average monthly admission rate increased from 2,577 in the April lockdown phase to 4,117 during the May to August period, whereas when expressed as a percentage of all admissions (i.e., a risk of the MDC 05 admission) it remained almost the same over the first two periods (11.90 and 12.14%, respectively).

Concerning the MDC 09 group, it amounted to approximately 3% of total monthly admissions, with a somewhat higher percentage of monthly admissions during the May to August period (3.27%) as compared to that of the September to December period (2.82%).

Table 5 shows ranked, from highest to lowest percentage, MDCs during the Lockdown Phase (April 2020) compared with ranked MDCs during Period 1 (May to August 2020). It can be seen that the rank for the top ranking MDC admissions changed between the two periods in that the three top MDCs during the April lockdown phase were 14, 05, and 06, whereas during the May to August period the highest percent admission was for the 05, 14, and 08 groups. For each MDC group, both unadjusted/raw and multiplicity-adjusted *p*-values (for testing the hypothesis of no change over the two time periods) are presented in the last two columns. For example, MDC 14 ranked second in Period 1 with a percentage admission of 11.63% was significantly different (*p* < 0.0001) than MDC 14 during the April lockdown phase, where it ranked first with a percentage of 15.42%. However, the percent admissions of 3.27% for the MDC 09 group during the May to August period is not statistically different (multiplicity adjusted *p* = 0.4496) from the percent admissions during the April lockdown phase of 2.95%.

**TABLE 5:** Ranked MDCs during lockdown phase (April 2020) compared with ranked MDCs during Period 1 (May to August 2020).

April lockdown phase			Period 1 (May to August 2020)			Raw prob	Prob^a^
Rank	MDC class	Percent	Rank	MDC class	Percent		
1	MDC 14	15.42	1	MDC 05	12.14	0.3997	1.0000
2	MDC 05	11.90	2	MDC 14	11.63	<0.0001	<0.0001
3	MDC 06	9.92	3	MDC 08	11.23	<0.0001	<0.0001
4	MDC 04	9.20	4	MDC 06	10.05	0.6369	1.0000
5	MDC 08	8.11	5	MDC 01	8.11	0.8483	1.0000
6	MDC 01	8.07	6	MDC 04	5.88	<0.0001	<0.0001
7	MDC 11	5.37	7	MDC 11	5.22	0.4439	1.0000
8	MDC 07	4.74	8	MDC 07	5.10	0.0566	0.6797
9	MDC 17	4.02	9	MDC 13	3.41	<0.0001	<0.0001
10	MDC 09	2.95	10	MDC 17	3.37	<0.0001	0.0012
11	MDC 19	2.67	11	MDC 09	3.27	0.0346	0.4496
12	MDC 18	2.47	12	MDC 03	2.76	<0.0001	<0.0001
13	MDC 15	2.29	13	MDC 10	2.46	<0.0001	<0.0001
14	MDC 03	2.07	14	MDC 19	2.34	0.0133	0.1860
15	MDC 13	2.02	15	MDC 18	2.30	0.2086	1.0000
16	MDC 23	1.49	16	MDC 02	2.29	<0.0001	<0.0001
17	MDC 10	1.40	17	MDC 15	1.93	0.0042	0.0670
18	pre	1.15	18	MDC 12	1.34	<0.0001	0.0010
19	MDC 16	1.03	19	MDC 23	1.21	0.0056	0.0834
20	MDC 12	0.96	20	MDC 16	1.09	0.4737	1.0000
21	MDC 21	0.86	21	pre	1.02	0.1339	1.0000
22	MDC 02	0.85	22	MDC 21	0.87	0.8928	1.0000
23	MDC 20	0.69	23	MDC 20	0.61	0.2626	1.0000
24	Error DRGs	0.26	24	Error DRGs	0.27	0.8280	1.0000
25	MDC 22	0.10	25	MDC 22	0.08	0.4654	1.0000

^a^The multiplicity-adjusted *p*-value for testing the hypothesis that the MDC percent (“risk”) in April Lockdown Phase is equal to that in Period 1 (i.e., that an individual MDC risk ratio is equal to 1).

**FIGURE 4:**
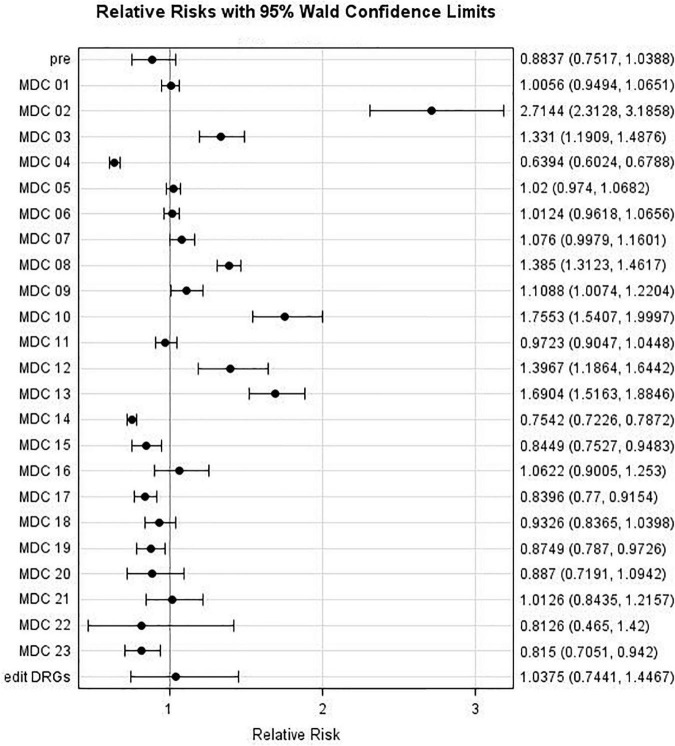
Major Diagnostic Categories (MDC) relative risk: MDC risk during Period 1 (May to August 2020) compared to MDC Risk during Lockdown Phase (April 2020).

Relative risks (RR) comparing individual MDC admissions during the May to August period with that during the April Lockdown Phase (with associated 95% confidence intervals) are displayed in Figure 4. It may be observed that the risk decreased for MDCs 04 and 14 [with Relative Risks (RRs) of 0.64 and 0.75, respectively], whereas it increased for MDCs 02, 03, 08, 10, 12, and 13 (with RRs of 2.71, 1.33, 1.39, 1.76, 1.40, and 1.70, respectively). For example, patients admitted to a hospital during the May-Aug period were almost three times as likely to be categorized as MDC 02 (eye) group as were those admitted during the April lockdown phase (*p* < 0.0001).

**FIGURE 5:**
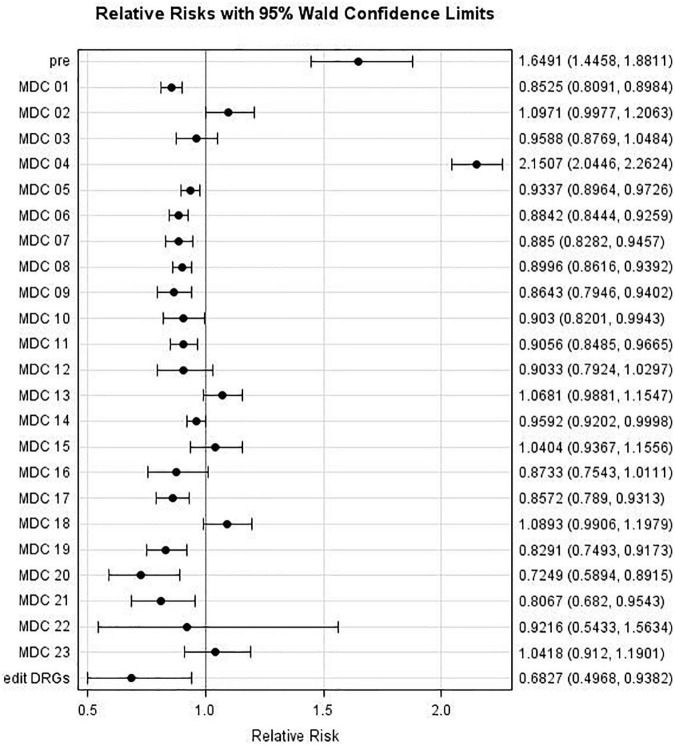
Major Diagnostic Categories (MDC) relative risk: MDC risk during Period 2 (September to December 2020) compared to MDC risk during Period 1 (May to August 2020).

Likewise, Figure 5 and Table 6 present results regarding comparisons between Period 2 (September to December) and Period 1 (May to August). The admission risk decreased for the following MDCs: 01, 05, 06, 07, 09, 11, 17, 19, and 20 (with RRs of 0.85, 0.93, 0.88, 0.89, 0.86, 0.91, 0.86, 0.83, and 0.72, respectively), whereas it increased for only “pre” and MDC 04, with RRs of 1.65 and 2.15, respectively.

**TABLE 6:** Ranked MDCs during Period 1 (May to August 2020) compared with ranked MDCs during Period 2 (September to December 2020).

Period 1 (May to August 2020)			Period 2 (September to December 2020)			Raw prob	Prob^a^
Rank	MDC class	Percent	Rank	MDC class	Percent		
1	MDC 05	12.14	1	MDC 04	12.65	<0.0001	<0.0001
2	MDC 14	11.63	2	MDC 05	11.34	0.0010	0.0159
3	MDC 08	11.23	3	MDC 14	11.16	0.0490	0.4899
4	MDC 06	10.05	4	MDC 08	10.11	<0.0001	<0.0001
5	MDC 01	8.11	5	MDC 06	8.88	<0.0001	<0.0001
6	MDC 04	5.88	6	MDC 01	6.91	<0.0001	<0.0001
7	MDC 11	5.22	7	MDC 11	4.73	0.0028	0.0394
8	MDC 07	5.10	8	MDC 07	4.51	0.0003	0.0055
9	MDC 13	3.41	9	MDC 13	3.64	0.0973	0.5836
10	MDC 17	3.37	10	MDC 17	2.89	0.0003	0.0054
11	MDC 09	3.27	11	MDC 09	2.82	0.0007	0.0115
12	MDC 03	2.76	12	MDC 03	2.65	0.3562	1.0000
13	MDC 10	2.46	13	MDC 02	2.52	0.0558	0.5023
14	MDC 19	2.34	14	MDC 18	2.51	0.0774	0.5583
15	MDC 18	2.30	15	MDC 10	2.22	0.0377	0.4148
16	MDC 02	2.29	16	MDC 15	2.01	0.4598	1.0000
17	MDC 15	1.93	17	MDC 19	1.94	0.0003	0.0054
18	MDC 12	1.34	18	pre	1.68	<0.0001	<0.0001
19	MDC 23	1.21	19	MDC 23	1.26	0.5461	1.0000
20	MDC 16	1.09	20	MDC 12	1.21	0.1277	0.6385
21	pre	1.02	21	MDC 16	0.96	0.0698	0.5583
22	MDC 21	0.87	22	MDC 21	0.70	0.0121	0.1569
23	MDC 20	0.61	23	MDC 20	0.44	0.0022	0.0331
24	Error DRGs	0.27	24	Error DRGs	0.18	0.0179	0.2150
25	MDC 22	0.08	25	MDC 22	0.08	0.7621	1.0000

^a^The multiplicity-adjusted *p*-value for testing the hypothesis that MDC percent (“risk”) in Period 1 is equal to that in Period 2 (i.e., that an individual MDC risk ratio is equal to 1).

## Discussion

### Observations

During the pandemic, there was a significant average decrease (−21%) in the total number of admissions and (−29%) in the number of hospitalized dermatological patients at both secondary and tertiary hospital levels. The average decrease of hospitalized dermatological patients was greater for 2020 than 2021 (32% vs. 26%) compared to 2017–2019. On February 25, 2020, the first patient with COVID-19 was identified in Croatia. Three weeks later, hospital care delivery was modified to meet the assessed needs of the pandemic in response to an increased COVID-19 patient load and escalating danger of contagion. Three hospitals in Zagreb, the capital city of Croatia, were designated as COVID-19 centers, and patients with COVID-19-related illnesses requiring inpatient care were admitted to those facilities. The majority of big hospitals set up COVID-19 isolation ward, and four comparable facilities were established around the regions (17). In addition to the restructuring of the healthcare system, 2020 also saw tighter controls (lockdown). Hospital staff shortages, reprioritization of elective operations by hospitals, and a fall in the non-emergency admission referral rate as a result of fewer outpatient hours are further contributing causes. Fear of acquiring COVID-19 infection in a hospital setting is another (18). Also, in March 2020, Zagreb faced another disaster - a devastating earthquake that destroyed some hospitals and Dermatology departments. The less average decrease in 2021 may be explained by milder measures, weaker restrictions, and the start of vaccination against COVID-19. The decrease in 2021 is still statistically significant. In 2020, a majority of procedures were postponed, because patients admitted for COVID-19 and non-COVID-19 emergencies had prioritized treatment. In 2021, hospitals have started to accept non-COVID-19 patients.

Kutlu et al. reported similar results in a study conducted in Turkey. Following the COVID-19 epidemic, there was a rapid decline in the number of people seeking appointments at dermatology outpatient clinics. They discovered a significant inverse relationship between the number of patient cases and COVID-19-related mortality and the number of patients asking to visit an outpatient dermatology clinic. Requests for dermatological outpatient clinic appointments fell as the number of COVID-19 cases and deaths associated with them rose (19).

Dang et al. (20) conducted similar study about impact of COVID-19 on inpatient admission changes at National Hospital of Dermatology and Venereology in Vietnam. In 2020 and 2021, respectively, the number of patients significantly dropped by around 30 and 60%. The most significant increase from 5.9% in 2018 to 21% in 2021 was in benign neoplasia. The allergic/reactive group dropped from 40% in 2018 to 25% in 2021. Additionally, the infectious group dropped from 20% in 2018 to 10.8% in 2021 (20).

In Australia, there was also 16% reduction in dermatology consults in 2020 compared with 2018 (21).

During the pandemic, there was also a significant decrease in surgical procedures performed on dermatological patients. Due to the possibility of contamination, cosmetic and non-urgent surgical operations were not performed. Dermatological conditions can raise the risk of infection in patients because they can make it easier for viruses to spread through indirect contact (22). We found that the average decrease in surgical procedures was 22%. A higher decrease, 25% was at the tertiary hospital level, compared to 17% at the secondary hospital level. Almost all surgical procedures, except major breast reconstruction, faced negative rate change. Major breast reconstruction has an average rate change of 195%, 206% at the tertiary, and 67% at the secondary hospital level. Patients with cancer were prioritized in hospitalization and treatment during the pandemic because the risk of postponing surgery and further treatment in oncologic patients was high.

The study from Germany (23) revealed similar results, with an average decrease of 17% in skin cancer surgical procedures (23). In Italy, it was observed a 30% decrease in surgical activity during the first lockdown (24).

The reduction in non-surgical treatment of dermatological conditions was also present in all categories, except J62B (*p* = 0.488), and J68B (>0.9999). The average decrease was by 37%, at the tertiary hospital level by 36%, and at the secondary level 39%. The highest decrease in hospitalization was noted in patients with cellulitis, trauma to the skin, subcutaneous tissue and breast, and major skin disorders.

Białynicki-Birula et al. discovered that in Poland had been a considerable drop in the number of patients being admitted with the following diagnoses: atopic dermatitis, various eczematous dermatoses, lichen planus, and pityriasis rubra pilaris. As opposed to this, patients with erysipelas, syphilis, and primary cutaneous lymphomas were admitted at a rate that was noticeably greater in 2020 than it was in 2019 (25). In 2020, in Italy, reduced admission to outpatient clinics and day hospitals was seen in patients with rare skin disorders (26).

Early in the COVID-19 pandemic, a sharp decline in the number of skin biopsies was seen; this adversely affected women, the elderly, and people who lived in particular areas. The Delay biopsies persisted long after the initial lockdown measures were implemented. This will have an impact on skin cancer treatment in the future (27).

Diseases of the respiratory system (MDC 04) show different trends compared to other MDC groups. In MDC 04 the average monthly admission rate was relatively stable over the April and May-Aug phases but then increased during the September-December period. Patients admitted to a hospital during the September-December period were more than two times as likely to be categorized in the MDC 04 (respiratory system) group as those admitted during the May to August period of 2020. This trend may be explained by different measures and restrictions in those periods–lockdown and strict restrictions in April 2020, milder restrictions in the summer period, and the second wave of COVID-19 and colder weather which is usually linked with a higher incidence of respiratory diseases.

### Strengths and limitations

The main strength of this study is the utilization of a full data set on inpatient activity for all Dermatology departments in Croatia. It also describes the impact of the SARS-CoV-2 pandemic on the number of hospitalized patients with dermatological diseases, as well as the number of patients treated surgically and conservatively, at the level of secondary and tertiary health care. The limitation of this research is that we did not have available private providers data to compare.

## Conclusion

Coronavirus disease 2019 had a great impact on inpatient care related to skin conditions. It is expected that the reduced availability of health care during the pandemic will result in a later diagnosis of the disease, delayed start of treatment, a worse outcome of the disease, and also increased costs of health care. Teledermatology is still not widely used, but the experience of the past 2 years further suggests that it could have a place in care for patients with skin diseases.

## Data availability statement

The raw data supporting the conclusions of this article will be made available by the authors, without undue reservation.

## Ethics statement

Ethical review and approval was not required for the study on human participants in accordance with the local legislation and institutional requirements. Written informed consent from the patients was not required to participate in this study in accordance with the national legislation and the institutional requirements.

## Author contributions

KK, AO, and MP led design of the study. KK organized the data collection and analysis of the first draft. RM, MŠ, and SO performed the statistical analysis of the data collected and wrote the findings section. All authors contributed to the development of the research question, study design in relation to the Croatian DRG hospital data, interpretation of the results, and critical revision of the manuscript for the important intellectual content and approved the final version of the manuscript.

## Footnotes

1 Pre MDC–Major procedures related to principal diagnoses belonging to any MDC; MDC 01–Diseases and Disorders of the Nervous System; MDC 02–Diseases and Disorders of the Eye; MDC 03–Diseases and Disorders of the Ear, Nose, Mouth and Throat; MDC 04–Diseases and Disorders of the Respiratory System; MDC 05–Diseases and Disorders of the Circulatory System; MDC 06–Diseases and Disorders of the Digestive System; MDC 07–Diseases and Disorders of the Hepatobiliary System and Pancreas; MDC 08–Diseases and Disorders of the Musculoskeletal System and Connective Tissue; MDC 09–Diseases and Disorders of the Skin, Subcutaneous Tissue and Breast; MDC 10–Endocrine, Nutritional and Metabolic Diseases and Disorders; MDC 11–Diseases and Disorders of the Kidney and Urinary Tract; MDC 12 -Diseases and Disorders of the Male Reproductive System; MDC 13–Diseases and Disorders of the Female Reproductive System; MDC 14–Pregnancy, Childbirth and the Puerperium; MDC 15–Newborns and Other Neonates; MDC 16–Diseases and Disorders of the Blood and Blood Forming Organs and Immunological Disorders; MDC 17–Neoplastic Disorders (Hematological and Solid); MDC 18–Infectious and Parasitic Diseases; MDC 19–Mental, Behavioral and Neurodevelopmental Disorders; MDC 20–Alcohol/Drug Use and Alcohol/Drug Induced Organic Mental Disorders; MDC 21–Injuries, Poisoning and Toxic Effects of Drugs; MDC 22 – Burns; MDC 23–Factors Influencing Health Status and Other Contacts with Health Services; Error DRGs.

2 J01Z–Microvascular Tissue Transfer for Skin, Subcutaneous Tissue and Breast Disorder; J06A–Major Procedures for Malignant Breast Conditions; J06B–Major Procedures for Non-Malignant Breast Conditions; J07A–Minor Procedures for Malignant Breast Conditions; J07B–Minor Procedures for Non-Malignant Breast Conditions J08A–Other Skin Graft and/or Debridement Procedures W Catastrophic or Severe CC; J08B–Other Skin Graft and/or Debridement Procedures W/O Catastrophic or Severe CC; J09Z–Perianal and Pilonidal Procedures; J10Z–Skin, Subcutaneous Tissue and Breast Plastic OR Procedures; J11Z–Other Skin, Subcutaneous Tissue and Breast Procedures; J12A–Lower Limb Procs W Ulcer/Cellulitis W Cat CC; J12B–Lower Limb Procs W Ulcer/Cellulitis W/O Cat CC W Skin Graft/Flap Repair; J12C–Lower Limb Procs W Ulcer/Cellulitis W/O Cat CC W/O Skin Graft/Flap Repair; J13A–Lower Limb Procs W/O Ulcer/Cellulitis W Skin Graft W (Cat or Sev CC); J13B–Lower Limb Procs W/O Ulcer/Cellulitis W/O [Skin Graft and (Cat or Sev CC)]; J14Z–Major Breast Reconstructions.

3 J60A–Skin Ulcers; J62A–Malignant Breast Disorders (Age > 69 W CC) or W (Cat or Sev CC); J62B–Malignant Breast Disorders (Age > 69 W/O CC) or W/O (Cat or Sev CC); J63Z–Non-Malignant Breast Disorders; J64A–Cellulitis Age > 59 W Catastrophic or Severe CC; J64B–Cellulitis (Age > 59 W/O Catastrophic or Severe CC) or Age < 60; J65A–Trauma to the Skin, Subcutaneous Tissue and Breast Age > 69; J65B -Trauma to the Skin, Subcutaneous Tissue and Breast Age < 70; J67A–Minor Skin Disorders; J67B–Minor Skin Disorders, Sameday; J68A–Major Skin Disorders; J68B–Major Skin Disorders, Sameday.
